# “We have been depriving them”: examining the sense of coherence of clinical staff as they implement skin-to-skin contact

**DOI:** 10.3389/fgwh.2025.1595266

**Published:** 2025-07-25

**Authors:** Kajsa Brimdyr, Scovia N. Mbalinda, Anna Blair, Karin Cadwell

**Affiliations:** ^1^ Center for Breastfeeding, Healthy Children Project, Inc., Harwich, MA, United States; ^2^ Department of Nursing, College of Health Sciences, Makerere University, Kampala, Uganda

**Keywords:** skin-to-skin contact, salutogenesis, meaningfulness, relationships, maternity care

## Abstract

**Background:**

Skin-to-skin contact (SSC) immediately after birth, when the newborn baby and mother remain together during the first hour after birth, has positive health effects on the dyad's physical and emotional wellbeing; however, implementation, the purview of the hospital's labor and birthing unit staff, has been a challenge in many settings.

**Objective:**

To investigate Antonovsky's salutogenic theory's sense of coherence (SOC) of birthing staff members before and after implementing skin-to-skin contact immediately after birth in a regional referral hospital in Uganda.

**Method:**

This qualitative study explored and analyzed before-and-after interviews of clinical staff regarding their experience of practice change to immediate, continuous, and uninterrupted skin-to-skin contact for at least the first hour after birth. The semistructured interviews took place at a regional referral hospital in Western Uganda. Using thematic analysis, the interviews were analyzed for the three components central to SOC: whether the proposed change in practice (pre-SSC intervention) and experience of the change in practice (postintervention) was comprehensible, manageable, and meaningful.

**Results:**

An analysis indicated a high level of SOC before the intervention in relation to the meaningfulness and comprehensibility of SSC, with concerns about manageability. An analysis of postintervention interviews indicated a high level of SOC for all three aspects.

**Conclusion:**

We postulate that a high level of sense of coherence for hospital staff both before and after an intervention may play a role in successfully implementing immediate, uninterrupted skin-to-skin contact in the first hour after birth. Skin-to-skin contact immediately after birth has life-long consequences for the emotional wellbeing of both the mother and the newborn.

## 1 Introduction

Childbirth is not an illness or disease. It is not a pathology. Therefore, salutogenesis, Antonovsky's theory of health that examines factors that contribute to one's wellbeing (1), is optimally applied to hospital practices (2) including those related to pregnancy, birth, and postpartum for both patients (3–5) and staff (6). Skin-to-skin contact (SSC), where the newborn baby and the mother remain together during the first hour after birth, has been shown to have positive, salutogenic effects on the wellbeing of both the mother and the newborn (7). SSC occurs during a “sensitive period” for the dyad (8, 9). Immediately after birth, the baby has high levels of catecholamines. At the same time, there are high oxytocin levels in the mother (10), which are associated with maternal bonding and attachment (11–14). These two factors combine for a unique and crucial “sensitive moment” for bonding and have a relationship with a “vital importance of contact and touch” between the mother and the newborn (15). With SSC playing a role in stress regulation, this sensitive period of time has been linked to a sense of happiness, connection, and increased positive feelings in the mother (16). SSC may relieve post-traumatic stress, even in women who have had traumatic birth experiences (17). Early SSC has been linked to a decreased risk of early maternal depression and bonding problems (18), and reduced maternal anxiety (19). Stress levels, as measured by salivary cortisol, a key biomarker of stress, are significantly reduced after childbirth in women who have experienced SSC (20). These advantages to the mother are also relevant after cesarean surgery, with research demonstrating that SSC is linked to decreased maternal stress and increased comfort, oxytocin, and levels of antioxidants (11). These, and other positive outcomes for mothers and their babies, have encouraged clinicians and policymakers to implement and scale-up SSC worldwide.

In spite of inclusion in WHO/UNICEF's Baby-Friendly Hospital Initiative (Step 4) (21), practice implementation of SSC remains low. A systematic review of the prevalence of SSC throughout the world, including 28 countries in all six WHO world regions, suggests a wide range of practice, from 1% to 98% (22). Only 15 articles of the 35 in the review defined SSC, so it is unclear whether these practices included the WHO recommendation of immediate, continuous, uninterrupted SSC for at least the first hour after birth. Challenges to implementing SSC include a lack of motivation and skills (23) of the healthcare providers as well as staffing concerns, time limitations, concerns about potential adverse effects, and the impracticality of the duration of 1 h (24).

Prevalence of skin-to-skin contact in Uganda is reported to be high, 73%. However, the question asked in the survey: ‘Was child put on mother's chest and bare skin after birth?’ does not include the specifics of the international definition; it does not elaborate on whether the SSC was immediate, continuous, and uninterrupted SSC for at least an hour after birth. The 2023 International Guidelines on skin-to-skin contact in the first hour after birth (25) has the potential to provide a roadmap to the Ugandan Ministry of Health's goal of decreasing maternal and infant mortality (26) by increasing best practices in SSC. However, the responsibility of implementation falls on clinicians—midwives, medical interns, and medical doctors who work in the maternity units.

Health and wellbeing are prioritized in Antonovsky's salutogenic theory. Sense of coherence (SOC) forms the core construct of the model and focuses on the capacity of the individual, family, or community to use resources in the environment to maintain and improve health, even in the face of a stressful event or challenge. It is comprised of three components: comprehensibility, manageability, and meaningfulness (1). Comprehensibility refers to the idea that stressful factors are structured, predictable, explainable, consistent, and clear. Manageability refers to the idea that adequate resources are available to cope with stressful factors. Meaningfulness refers to the idea that a challenge is worthy of investment and engagement, and worthy of commitment and involvement. A person with a strong SOC, when faced with a stressful emergency, crisis, or disease, will be able to incorporate the elements of SOC to understand, make meaning of, and manage the situation more successfully than one who has a more fragile SOC. Generalized Resistance Resources (GRRs) are characteristics that help an individual cope with stressors. GRRs can range from material resources to religious beliefs to genetic makeup. Conceptual expansions of these theories have included families, communities, and workplaces as well (27).

According to Antonovsky, work itself needs to be comprehensible, manageable, and meaningful if it is to be considered salutogenic (28, 29). The workplace can be a source of both stress and pleasure, and so can influence an individual's wellbeing both positively and negatively. Antonovsky's line of thinking can be applied in a wider scope then originally intended, by including the workplace itself as a community. It would then follow that the workplace can have an SOC and GRRs, in this case, referred to as job resources (28).

In relation to the practice of providing skin-to-skin in the first hour after birth, thematic analysis using SOC as a lens allows for an examination of the staff's capacities to understand, motivate, and use available job resources to maintain and improve staff work practices and the environment. The first dimension of SOC, comprehensibility, focuses on a clear and comprehensive understanding of the implementation of skin-to-skin contact in the first hour after birth. Along with the protocol and the practice, this cognitive component includes an understanding of how to deal with stressful events that could occur (30).

The next dimension of SOC, manageability, is considered to be a behavioral component (30). It should effectuate an underload–overload balance that takes resources into account. Resources, in this case, job resources, could include those directly under staff control, or resources controlled by others. In relation to skin-to-skin in the first hour after birth, resources controlled by others could include daily staffing in the ward, the space and layout of the ward, and training provided by the management. Resources controlled by the staff include patient access to the ward and the use of the space. Stressors should be manageable; demands and resources should be in balance. Underload can lead to a lack of engagement. Overload can lead to stress and burnout. Job dissatisfaction and burnout, which are considered modifiable states, are associated with increased rates of missed maternity care (31). SOC, among other effects, “*influences* work related outcomes, such as burnout and stress symptoms” (28, p. 199). Changing clinical practices can be perceived as a stressor in staff.

The third dimension of SOC, meaningfulness, is considered to be the motivational component (30). It focuses on the idea that if a challenge is worthy of engagement, then it is worth the investment of resources. Research indicates that happiness has an impact on productivity and job satisfaction (32). Skin-to-skin contact is an acknowledged pathway for connectiveness between parents and newborns; a means to increase bonding (13) with advantages for parents and newborns, including lower stress levels in the mother and the baby (33). Although it is assumed that the advantages of SSC for the mother and baby would be meaningful to the staff, research has not been published about this aspect of SSC.

Introducing practice change, in this case the implementation of immediate, continuous, and uninterrupted SSC can be considered a workplace stressor. Barriers have been described that thwart SSC implementation, including a lack of staff, time, and training (23, 24, 34). Implementation of a practice change could be considered a “job demand” within Antonovsky's model of salutogenesis in the context of work (28). The implementation of a new practice can be buffered by the GRRs, which in this context are the job resources, such as knowledge, skill, social support, and autonomy.

Although earlier studies have mostly focused on SSC's therapeutic results, fewer studies have addressed the human elements affecting its application. Although this association has been understudied, understanding how the elements of SOC can affect the implementation process could be relevant. Examining SOC in this framework might help explain why some teams struggle, while others effectively incorporate SSC in spite of seemingly similar constraints.

This qualitative study aimed to analyze semistructured interviews of clinical staff through the lens of the three elements of SOC before and after the implementation of a new practice model of postpartum care.

## 2 Materials and methods

The study was conducted at a regional referral and training hospital in western Uganda, between January and February 2024, which averages 15–20 births a day. The methodology and outcomes of the intervention, which involved the implementation of immediate, continuous, and uninterrupted skin-to-skin contact, have been reported elsewhere (35). Semistructured interviews with leadership and staff, conducted by research team members, were audio-recorded before the intervention and toward the end of the postintervention birth cohort collection. The questions were open-ended and focused on the barriers to implementing the new practice (Table 1). The six preintervention key informants included one medical intern, four midwives, and one midwife in a senior leadership position.

**Table 1:** Key informant interview questions.

Preintervention questions
What do you know about mothers holding their babies skin-to-skin in the first hour after birth?
Do you think it is possible to have this practice for vaginal births at this hospital? Why or why not?
Do you think it is possible to have this practice for cesarean births at this hospital? Why or why not?
What do you think will be the biggest challenges to starting skin-to-skin in the delivery room for your hospital?
What do you think will be the biggest challenges to starting skin-to-skin in the operating room for your hospital?
Postintervention questions
What do you know about mothers holding their babies skin-to-skin in the first hour after birth?
Do you think it is possible to continue to have this practice for vaginal births at this hospital? Why or why not?
Do you think it is possible to continue to have this practice for cesarean births at this hospital? Why or why not?
What do you think will be the biggest challenges to continue skin-to-skin in the delivery room for your hospital?
What do you think will be the biggest challenges to continue skin-to-skin in the operating room for your hospital?
Can you share some of your experiences with skin-to-skin at your hospital?

The full intervention methodology, Practice Reflection Education and Training Combined with Ethnography for Sustainable Success (PRECESS), is described elsewhere (35). In short, an educational session for leadership and staff took place after the preintervention data collection was completed. Participants included midwives, doctors, surgical technicians, interns, nurses, nursing and midwifery students, as well as admissions staff. The room held 20 people, and additional staff stood in the doorway and in the hallway for portions of the presentation. The researchers used PowerPoint presentations and videos to demonstrate the justification and practice of keeping babies SSC for 1 h after birth as well as videos of newborns progressing through Widström's nine stages, self-attaching and suckling. The change in practice to 60 min of SSC began with the birth that occurred immediately after the education session. Following the PRECESS protocol, the researchers were available 24 h a day to provide pragmatic and practical assistance as well as support during the intervention. This could include answering questions, providing recommendations, demonstrating positioning, etc. The quantitative analysis of this study is reported elsewhere (35). The intervention significantly increased the duration of SSC from a mean of 2 min 25 s before the intervention (92 dyads over 7 days) to a mean of 57 min 51 s after the intervention after (105 dyads over 7 days) (*p* < 0.001).

The second set of key informant interviews were conducted 6–7 days after the initiation of the practice change. The postintervention key informants included two medical interns, three midwives, one obstetrician, and one midwife in a senior leadership position. Only one key informant, the midwife in the senior position, had also participated in the preintervention interviews. The recorded interviews were transcribed and analyzed using the theoretical thematic analysis method suggested by Braun and Clarke (36). Following Braun and Clarke's six phases, the transcripts were analyzed by three researchers familiar with the process. After familiarizing themselves with the data (transcripts), they developed initial codes to identify the three aspects of SOC. Transcripts were then coded for the themes of the three components of SOC, and initial theoretical thematic analyses completed. Text that expressed an understanding or misunderstanding of the practice of SSC was coded for “comprehensibility.” This included concepts around the structure and predictable nature of the process of SSC. Text that related to the capability of implementing one hour of SSC and whether job resources were enough to make and sustain the change to extended SSC were coded for “manageability.” This included staffing, space, and physical resources. Text that identified the value of SSC, a sense of purpose related to the change, or finding meaning in the practice was coded as “meaningfulness.” This included engagement and commitment to SSC. Analyses were discussed. All investigators reached consensus regarding the aspects of SOC and the transcript elements to include as illustrative.

Ethical approval was obtained from Makerere University College of Health Sciences, the School of Health Sciences Institutional Review Board (IRB) (MAKSHSREC-2023-558), and the Uganda National Council for Science and Technology (UNCST) (HS3183ES) prior to data collection. The study received administrative clearance, informed consent, and voluntary participant participation, ensuring confidentiality and respecting participant privacy. Audiotapes and notes were kept securely.

## 3 Results

Quotes from the interviews have been categorized into the three focal points of SOC: comprehensibility, manageability, and meaningfulness. In each case, the interviews preintervention and postintervention have been examined for aspects of each focal point.

### 3.1 Comprehensibility

Comprehensibility is the understanding that a process, specifically skin-to-skin contact in the first hour after birth, is predictable and structured. The comprehensibility of the practice of SSC, both before and after the intervention, has been contextualized by other clinical parameters.

#### 3.1.1 Preintervention: comprehensibility

All six of the preintervention interviews expressed a clear and comprehensive understanding of the current practice of skin-to-skin contact (baby placed on the mother's abdomen before the cord was clamped, and removed to a table after clamping, around 2–3 min of SSC, on average).

“I would put a sheet [on the mother], dry the baby, then after drying and I am sure the baby is not wet any more, if it does not need any other resuscitation and the baby is fine, I put the baby on the mother’s tummy with the head-turned on one side then I cover the baby with the sheet on the mother’s abdomen for 2–3 min as I tie the cord … and after clamping the cord I take the baby away” –(Midwife 2).

This quote emphasized confidence in the structured current practice, even though current international recommendations recommend one hour of skin-to-skin contact, rather than the current practice of removing the baby after only 2–3 min. The staff had a high level of comprehensibility of the preintervention practice. This also reflected the staff's autonomy with the current practice.

When asked about challenges to the proposed intervention of 1 h of SSC, four key informants presented hypothetical stressful events that they saw as challenges. One key informant considered repairs a hindrance to continued skin-to-skin contact.

“Another challenge may be skin-to-skin, especially if a mother has gotten a perineal tear when you are repairing … it is because of the pain the baby feels” –(Midwife 6).

Because more than 85% of mothers experience perineal tears after vaginal birth (37), this demonstrated a significant concern related to the practice to 1 h of SSC. Perineal tear repairs are painful and could require significant suturing with limited anesthesia. Reactions from the mother could result in unexpected movements, which could not be conducive to SSC.

One key informant expressed concern about life-threatening events for the mother and the baby precluding the ability to do continued skin-to-skin contact.

“The challenges … [if] mother is bleeding and if this baby is asphyxiated, we cannot practice that [skin-to-skin]. But when I see my mother is okay, and my baby is okay, I do not see the reason why I cannot leave the baby to bond …” (Midwife 3).

Concerns about the mother and the baby's survival demonstrated a clear and comprehensive understanding of a stressful factor in Ugandan maternity units. Uganda's maternal mortality rate, 189 out of 100,000 births, is still significantly higher than the WHO goal of 12 per 1,000 (122 out of 100,000). It is estimated that 50% of neonatal deaths stem from birth asphyxia, with a current rate of 22/1,000 live births (38).

A third key informant expressed the lack of comprehensibility for a process for which they were not familiar. Without the comprehensibility for a new process, theoretical challenges create stressful factors. This key informant, who did not work in the operating theater, felt that a challenge for skin-to-skin contact during a cesarean section surgery would be the drapes.

“Even that cloth that prevents you from seeing what is being done there, how will you help the baby? You will just feel, but you cannot see the baby” –(Midwife 2).

This quote highlighted a misunderstanding about where the baby would be placed on the mother's chest during a cesarean operation in relation to the placement of the drape.

This same midwife also expressed concern for first-time mothers, who would need to try to breastfeed their baby within an hour after birth (a hospital protocol) in the uncomfortable position of lying on their back or lying on their side on a narrow delivery bed.

“… within that one hour, like for mothers who are delivering [for] their first time, have never breastfed these babies … they cannot breastfeed the baby when lying like this (on their back) and the baby is breastfeeding on [their] side.”

Again, this quote highlighted a misunderstanding about the positioning for breastfeeding during SSC, and that the position is conducive to breastfeeding in the first hour after birth.

#### 3.1.2 Postintervention: comprehensibility

All seven of the postintervention interviews expressed a clear and comprehensive understanding of immediate skin-to-skin for the full hour after birth. The process was comprehensible and also reflected the autonomy of the staff with the process.

“After delivery, we usually bring our baby to the skin, that is, to the mother’s chest. That one we provide it for approximately an hour to provide benefits to the mother and the newborn” (Intern 9).

This practice reflected the WHO standard of care for the first hour after birth.

The advantages were also clear.

“For the benefits, there is warmth. It helps to prevent hypothermia. It helps bonding, that is, for the mother to bond better and probably study the baby better, as well as their movements and whatever they signal to the mother. Then, it helps to reduce the risk of infection because when we take these babies away from their mothers, we expose them probably to an environment which has been exposed to by some other babies, which is also contaminated” (Doctor 15).

This reflected an evidence-based understanding of the scientific background knowledge underpinning SSC.

When asked about challenges, four key informants presented hypothetical stressful events that they saw as challenges. They mentioned three events that would preclude immediate, continuous skin-to-skin contact. The first concern was general anesthesia cesarean section surgeries, although this key informant agreed that the spinal surgeries conducted so far were comprehensible.

“[It] still depends on the anesthesia … We have so far practiced [SSC] with spinal anesthesia, where the mother is allowed to be conscious, and she can control her body parts. So, I am also imagining a mother who is in general anesthesia and not even responding to any reflex, so in that case, it will be hard”- (Intern 9).

But another key informant had been reflecting on general anesthesia as well:

“For the part of spinal anesthesia versus general anesthesia … lately, we do not do a lot of general anesthesia; we don’t. Out of 10, if there are 10 caesarean sections to be done in a day actually, 10 of them might be all spinal anesthesia. But true, if it is general anesthesia it gets hard” (Doctor 15).

The primary type of anesthesia used in the OR was considered to be comprehensible for SSC. The team had not yet conducted SSC with the unusual anesthesia, general anesthesia, and so it was still considered concerning and a source of stress.

This same key informant also expressed concerns about multiple babies, specifically triplets, but explained that twins should not preclude SSC.

“But if you have more than two, we usually deliver triplets here; if not, it becomes impossible. Otherwise, it can be achieved the other way around” (Intern 9).

Immediate, continuous, and uninterrupted skin-to-skin contact after birth for twins was considered to be comprehensible, implying that singletons were also considered comprehensible. The hypothetical concept of three babies on the mother's chest remained questionable to the key informant.

Intern 9 also expressed concerns about doing SSC after unusual incision types for cesarean surgeries.

“We have practiced [SSC] with Pfannenstiel incisions. I am thinking of a situation where I am doing subline umbilical incisions” (Intern 9).

Doing SSC after the most routine method of incisions for cesarean surgery was considered comprehensible. The concern voiced reflected concern about where a baby would be placed after unusual types of cesarean incisions.

Key informants mentioned specific elements about the explicability and predictability, including the impact of their routine medications:

“Some of the factors related to the health providers and the way we dispense [Misoprostol] could also lead to the frustration or failure of a peaceful skin-to-skin” (Intern 8).

The most common side effects for high doses of misoprostol, such as the dose routinely administered in the hospital to prevent postpartum hemorrhage, are fever and chills (shivering) (39), which can be disruptive to the process of SSC.

The newborn behavior while skin-to-skin after birth had also become comprehensible, including the understanding of the nine stages that newborns experience in the first hour after birth:

“It is nice when … you, the midwife who has helped with this delivery, see that within one hour, your baby goes through all the stages, the nine stages you have taken us through” (Midwife 14).

These instinctive behaviors when SSC is established in the first hour after birth lead to breastfeeding, a key advantage of SSC. The midwife was able to watch the baby while in SSC, to monitor the stages, and appreciate the success.

The structure and comprehensibility of the hour of skin-to-skin contact also had a positive effect on other procedures within the first hour, such as the effectiveness of repairing a tear during skin-to-skin contact.

“The process makes the mother calm when you are working on her in case of any tear” (Intern 8).

Research shows that SSC decreases the mother's perception of pain (10), which results in an easier experience for the staff to work on any repairs.

### 3.2 Manageability

Manageability, in relation to skin-to-skin contact, refers to the balance of adequate job resources available to cope with practice change. It is vital that sufficient consideration of resources is available in comparison with the demands so that SSC is manageable, neither overloaded nor underloaded.

#### 3.2.1 Preintervention: manageability

All six of the preintervention interviews expressed concern about the manageability of changing the process to skin-to-skin contact for an hour after birth. Four of the key informants mentioned concerns about staffing shortages.

“There was a day I was alone on duty. I had 18 deliveries in one shift” (Midwife 6).

“The staff is overloaded with work. Like today, I have come to work alone, so if I deliver a mother here, … I will leave them for another due to the inadequate staff” (Midwife 4).

This reflected a concern that implementing a full hour of immediate, continuous, and uninterrupted SSC would increase the time pressure on the staff to implement the practice and increase the amount of care needed for each dyad.

All six key informants mentioned concerns about the space and not having enough delivery beds.

“There are issues with the beds … once this one delivers, immediately we shift, and we take another mother” (Midwife 3).

There was a concern that leaving a mother in a bed for a full hour after birth to meet the WHO SSC standard would result in other mothers needing to give birth on the floor, since no beds would be available.

“Maybe the challenge can be if there are many mothers laboring, because of the space, as you leave this mother here skin to skin for one hour, another mother is immediately pushing and eventually going to push on the floor. Actually, what limits us here is space” (Midwife 6).

The key informants also expressed concerns about training about the practice of SSC.

“[A] good number of the staff, after employment, get little chances for exposure, like for these refresher trainings” (Midwife 6).

The key informant was concerned about learning a new skill and the need for continuing education after employment.

Several key informants also mentioned specific concerns related to the operating theater. These included existing inadequate staffing resources.

“In theater, there is no one who [can] resuscitate the baby … immediately when the baby is out … because we are trained to resuscitate babies, and we have to really be there …. Because of shortage and work overload in maternity, the one who was … the midwife in theater was removed and brought to cover the gap in maternity” (Midwife 6).

There was a fear that this could be worse if skin-to-skin is implemented, since the work load was already high. The fear concerned the emergency practice of resuscitating newborns, but also the more practical aspect of someone available to hold or watch the baby while in SSC on the operating table.

“But there has to be a nurse who holds the baby on the table because this hand is receiving IV fluids, and this hand might be, due to anesthesia, it might not hold the baby. But there should be someone to hold it. Sometimes, these babies can be jumpy and easily fall” (Intern 7).

If both mother's arms were not available to hold the baby, a staff member would be needed to support the baby so that it would not fall off the operating table while in SSC.

There were also concerns about physical artifacts, specifically the resources of sterile cloths to wrap around the newborn.

“In theater, the linen still is a challenge …. Because now in there, when they are operating, they have only one sheet to receive the baby, the theater sheet, which they only use because this one is for the baby” (Midwife 5).

The hospital traditionally provided sterile linen for use during the operation. The baby would be taken to the table and dried with a hospital sterile sheet. However, once the baby has been removed from the sterile field, they were wrapped in a cloth the mother brought from home. If the baby was in SSC with the mother, they could be considered to be in the sterile field, and therefore should be using a sterile linen provided by the hospital to be dried, and then another sterile linen provided by the hospital to be placed over the newborn while in SSC. This doubled the required resources for each baby.

There were some indications that there were adequate resources for the challenge of skin-to-skin for an hour after birth. Two key informants mentioned the positive resource of the interns and students.

“With the help of the students and the volunteers, we are building … moving on like that” (Midwife 6).

“If I have an assistant, what I usually do after tying and clamping the cord [is that] I take the baby away” (Midwife 2).

These job resources increase the manageability of the staffing concerns, since the students, volunteers, and assistants could help with additional work in the unit.

#### 3.2.2 Postintervention: manageability

After implementing immediate, continuous, and uninterrupted skin-to-skin contact, the concept of staffing seems to have been reframed. Although there still was not enough staff, the process of skin-to-skin contact for the hour after birth could make the work easier:

“… So, if at all you are alone it is easy for you to do skin-to-skin and actually you are not overwhelmed by not getting an assistant [a student] to take the baby [to] the other side and you can continue working on the mother as the baby is on skin to skin” (Midwife 12).

Postintervention, the number of staff, and the need for additional assistance, decreased, since the dyad could remain together, SSC, instead of the midwife having two patients in two locations.

The concepts of not enough space/not enough beds had been reframed as well by the staff:

“We have been having some mothers occupying these beds, though they are not in the second stage … we will have to see them ambulate … other than occupying the bed when you are not in the second stage and … give the opportunity to one who has delivered to have that skin-to-skin within the recommended time … making it appear as if space is not really enough” (Midwife 14).

Instead of feeling as though there were not enough beds, they had reframed the issue as needing to keep the mothers ambulating until they were ready to have a bed. This left more beds available for mothers to remain in SSC.

“It is just a matter of seeing who is a priority and whom I can continue talking nicely to as they are ambulating. Yeah, there are challenges; yes, we can do it. It is just space which is a challenge, but we can find a solution to it” (Midwife 14).

The space remained a challenge, but it was manageable.

Questions about training remained on the staff's list of manageability.

“You see the baby [close] to fall[ing] off the bed because [the mothers] support this baby over their chest and some of them are not given enough information during antenatal” (Intern 8).

“It needs real training for each and every health worker to have the safety of the mother and the baby and they must be safe in case of a fall” (Intern 8).

However, the requests for training were now specific training requests, rather than general concerns about SSC. Specifically, there was a concern about newborn babies falling off of the narrow delivery beds during SSC, and a request for assistance in problem-solving.

Specific concerns regarding the operating theater continue after the implementation. For example, there were still concerns about the sterile cloths for drying and covering the newborn. However, now it was being framed as a challenge that seemed manageable.

“[We] need some type of clothing [and] we sterilize it and we incorporate it as part of our practice” (Doctor 15).

“So, if we can provide small towels for cleaning and covering them and we don’t use the other bed sheets or the towels the mothers bring from home. I think it can help to improve sterility” (Intern 9).

By funding and providing small linens that could be included in the hospital's sterilization process, this challenge can be solved.

There were also still concerns about staffing, specifically associated with skin-to-skin in the operating theater.

“… Especially in our setting, where we have few staff members who can help you provide manpower” (Intern 9).

This is similar to the staffing concerns that were voiced by the labor and delivery team with vaginal births. However, unlike the key informant reports from labor and delivery, the issue still remained with the operating theater staff.

### 3.3 Meaningfulness

Meaningfulness in the context of Antonovsky's SOC and this research refers to the concept that skin-to-skin contact is worthy of investment and engagement.

#### 3.3.1 Preintervention: meaningfulness

All six of the preintervention interviews expressed a desire to do skin-to-skin contact.

“Babies with their mothers’ skin to skin … it brings a connection of the baby and the mother, it makes the baby know I am with my mummy, so it starts from the time of birth, and it brings that mother-child love, and then it also brings warmth” (Intern 7).

The key informants could list the advantages of SSC, even if they were only providing two to three minutes of SSC with the initial practice. The advantages of connection, love, and warmth were meaningful.

When asked about how long skin-to-skin should continue, concerns were raised. Skin-to-skin was meaningful for the first few minutes but seemed concerning after that.

“I think the baby shouldn’t exceed 30 min. On the mother’s abdomen, there are hypothermia issues … not as such because skin-to-skin is the best method for preventing hypothermia, but you know the mother is already [laying] in the blood, has been given some medication that can bring on shivering … in some cases, you feel the baby can easily fall off the mother’s abdomen” (Midwife 2).

Evidence recommends at least an hour of continuous, uninterrupted SSC beginning immediately after birth. Babies who are in SSC are warmer and are less likely to have hypothermia (10, 40), which is acknowledged by the staff. The other concerns related to extended SSC beyond the first 2–3 min included the ability to clean the mother and the bed, the effects of the anti-postpartum hemorrhage (PPH) medications, and the narrow delivery beds.

There was also concern that longer periods of time skin-to-skin may not feel meaningful to the mother.

“Delivering on the floor … skin to skin can be done, but for the mothers, she will not feel good … ‘I delivered, and I waited for one hour with my baby …’ she will feel she was not attended to … whereas there was something I was doing for her and she didn’t know” (Midwife 2).

Without an opportunity to provide antenatal education to the mothers, their first introduction to an hour of SSC would be potentially surprising. It was important to the staff to maintain a connection with the mothers and key informants expressed concern that the mother may be dissatisfied with the experience.

Concern was also expressed that longer periods of skin-to-skin may also not be meaningful to colleagues.

“Then maybe some of us have a bad attitude. I may come and start implementing it, but another may say ‘why’? You introduce it to someone else and say, ‘not me I have some more mothers to deliver’” (Midwife 5).

There was a question of whether all staff would consistently comply with the new practice of 1 h of SSC, and an emphasis on the importance of it being meaningful to everyone.

The work of the team in the labor and delivery unit, including the operating theater, was meaningful to the group.

“Doing [the births] with someone and seeing that it leads to success … And making decisions with the [doctors] … we always make decisions as a team” (Midwife 6).

This highlighted the teamwork and social support that was already in place before the intervention. This represented an important job resource (GRR).

#### 3.3.2 Postintervention: meaningfulness

All seven of the preintervention interviews expressed that skin-to-skin contact was meaningful to themselves and their patients. They saw SSC as meaningful to the mothers:

“I think they feel better, there [is] this calmness [the mothers] feel while their babies are with them, they are not worried. Because before, the moment we take the baby, they keep asking us, ‘Where is my baby … where is my baby?’ like their mind is where their baby is. But right now, I think they feel better with their baby” (Midwife 13).

The mothers knew where their babies were—with them while SSC—rather than with multiple babies on the table on the other side of the room. The key informant expressed that this makes mothers calmer.

The key informants saw SSC as meaningful for the babies.

“There are so many activities this baby does like lifting the head, looking for the breast … we have seen that it is very wonderful yes because we have been depriving the baby of the maternal love and bonding and keeping the baby away for some good minutes that [now] we [are] trying to help the mother in this first one hour” (Intern 8)

The staff recognized the instinctive behaviors of the newborn during SSC and the importance of the experience to the bonding of both the mother and the baby. The staff member also recognized their role in making this bonding experience possible for the dyad or not.

They saw SSC as meaningful to the staff, in terms of getting their work done.

“It is a good thing; it gives a lot of happiness to the mother. The mother feels so good when they are holding their baby on her chest, and it gives them a lot of happiness and joy. They even give you room to do other things; if you are delivering the placenta or closing incisions, they don’t mind; they are just looking at their babies” (Intern 9).

And they also saw SSC as meaningful to the staff, in terms of increasing their personal reward.

“Actually, since l started working with [this], I climb [into] the bed to sleep and sleep deeply because I am satisfied with what l have done. I leave the hospital … excited and satisfied with my job” (Midwife 14).

## 4 Discussion

**Figure 1:**
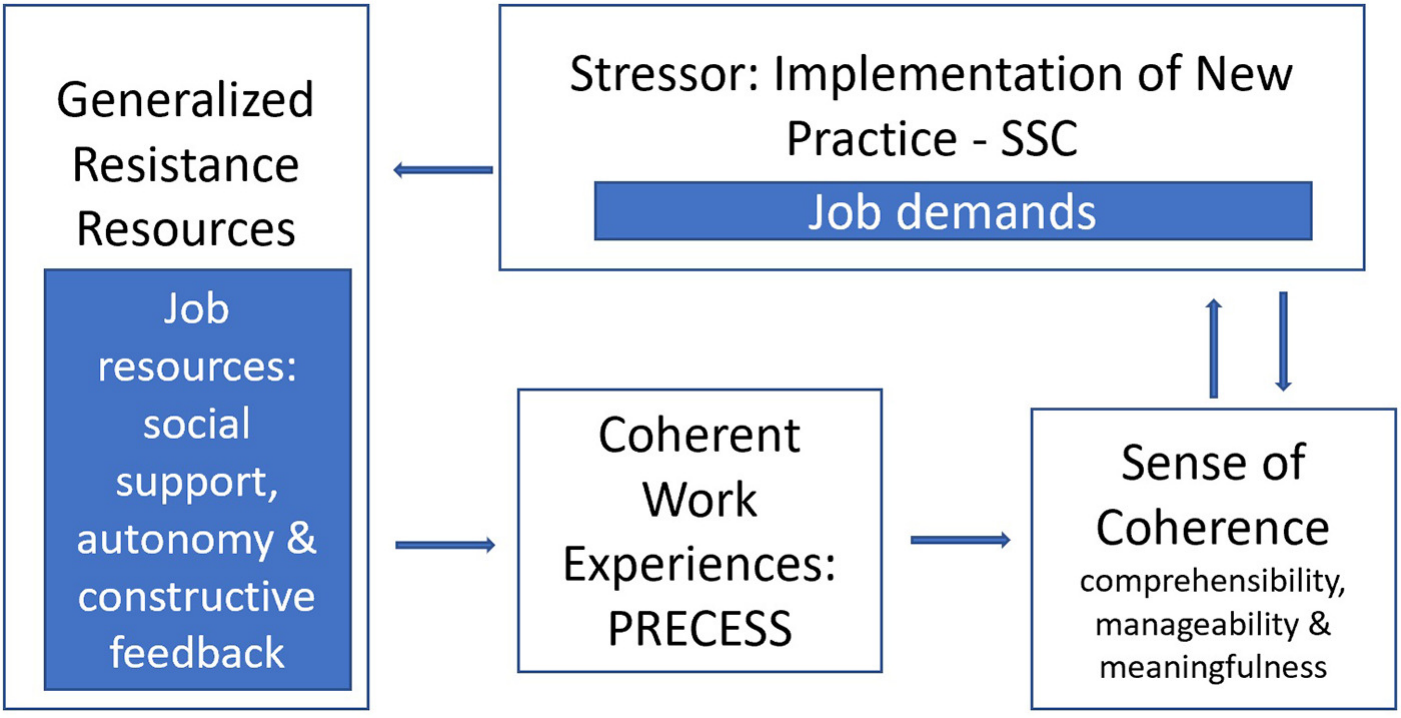
Applied model of salutogenic work practice for the context of implementing SSC. Adapted from Antonovsky (29).

PRECESS, a rapid practice change strategy that has been used to successfully implement SSC (35, 41), inherently creates a coherent work experience in that it supports a comprehensive, manageable, and meaningful practice transition. PRECESS does this through multiple means of providing job resources in the workplace: education (formal and informal) as well as 24 h pragmatic support, constructive feedback, problem-solving, and recognizing the existing skills and strengths of the team. However, this study is the first report of use of Antonovsky's SOC framework as the lens in a thematic analysis of staff members’ interviews before and after SSC practice change with the PRECESS strategy (Figure 1).

The staff exhibited high comprehensibility of their tasks related to SSC even before the intervention. However, the clinical practice changed from a few minutes of SSC to the international standard of immediate, continuous, and uninterrupted SSC for at least 1 hour. The resource concerns expressed in relation to the stressor/job demands of implementing a new practice changed. Before the intervention, the challenges that would preclude a full hour of SSC included common practices in the maternity unit or operating theater. After the intervention, challenges were still described. However, now the challenges expressed were for occurrences that were more hypothetical and unusual. SSC during the common events were not mentioned as challenges anymore, perhaps because all had been practiced and experienced during the intervention.

Before the intervention, the challenges to manageability of an hour of SSC immediately after birth included job resources such as staffing, training, and concerns about enough space. There were specific concerns about the OR, including the lack of sterile cloths to allocate to the newborn. After the intervention, the challenges of staffing were reframed, transforming SSC into a factor in increasing workplace manageability. Now, immediate, continuous, and uninterrupted SSC after birth helped the busy midwives, since the dyad remained together. This conceptual change in the meaning of the work is related to the crafting of the job and can contribute to a more salutogenic life (28). The issue of the manageability of the space was reframed as well. Now the midwives who were part of the key informant interviews reported that they had a justification to keep mothers ambulating until they were ready to deliver, instead of “taking” a bed too early. Walking during labor decreases the duration of the first stage of labor and also decreases the risk of cesarean (42). Implementing SSC had a serendipitous effect of increasing ambulation during labor. This, in turn, increased manageability for the staff. Concerns about the manageability of SSC in the OR continued, specifically the concern about a physical resource, sterile cloths.

Before the intervention, the two to three minutes of SSC was considered meaningful to the staff, although perhaps not for longer than it takes to cut the umbilical cord after it stops pulsing. The key informants expressed concern that the mothers and other colleagues would not value a longer experience of SSC. The key informants highlighted the meaningfulness of the staff cohesion and their teamwork in learning new skills. After the intervention, they saw SSC as meaningful for the mother and for the babies and the staff, both professionally and personally. Qualitative stories highlighted emotional rewards such as how the mothers would look at their babies and bond, that the babies were active and seemed more alert, and that their work felt more positive. This corresponds with studies showing nurse wellbeing to be correlated with perceived patient impact (43) and reflects Antonovsky's assertion that meaningfulness drives the “will to cope” (1). Burnout of staff is a concern in Uganda (44). Research has found that many midwives report work-related stress. Utilizing problem-solving has been found to be one coping technique for stress and burnout in healthcare work in other research in Uganda (45). Dealing with the stress of death and dying has been found to be a contributing factor of work-related stress for midwives in Uganda (44). Skin-to-skin in the first hour enhances factors associated with decreased maternal and neonatal death (10, 25). While preintervention, there were concerns about SSC leading to more stress for the staff; the result expressed by the key informants postintervention was the opposite, with comments such as feeling excited and satisfied after a long day of work.

### 4.1 Strengths and limitation

A strength of this study is the finding that success in the implementation of a new clinical practice may be influenced by the comprehensibility, manageability, and meaningfulness of the work of the staff and the job resources—in this case, the PRECESS team's 24 h/7 days a week availability. A limitation is that interview questions were not focused on specific SOC questions and did not elicit details of personal lives, history, and experiences, which could give insight into internal and external loads of individual staff members. An additional limitation is that this study reflects the implementation of SSC in one hospital in Uganda and may not be generalizable.

## 5 Conclusion

An analysis of the interviews with key informants through the lens of Antonovsky's SOC indicates that the hospital staff expressed comprehensibility, manageability, and meaningfulness related to their new practice of providing a full hour of immediate, continuous, and uninterrupted skin-to-skin contact after birth. Understanding and addressing these aspects of SOC when making practice change could have important implications on the uptake of SSC by practitioners and staff. Healthcare systems can turn SSC from a burden into a source of professional fulfillment by making investments in comprehensibility (education), manageability (resources), and by extension, meaningfulness (purpose of workplace tasks). The salutogenic approach ultimately reminds us that health is not the absence of difficulties but rather the capacity to negotiate them with coherence and hope—a lesson as important for caregivers as it is for patients. This has important salutogenic implications for mothers and babies as well, who can then benefit from the known advantages of this practice, including increased bonding, stability, and satisfaction. Implementing skin-to-skin contact, a worldwide priority in decreasing maternal and neonatal mortality, should consider the SOC of the staff when contemplating clinical practice change. Further research is needed to understand the implications of these findings in a variety of implementation strategies and settings.

## Data Availability

The original contributions presented in the study are included in the article/Supplementary Material, and further inquiries can be directed to the corresponding author.
